# Protection of wear resistance behaviour of enamel against electron beam irradiation

**DOI:** 10.1038/s41405-019-0021-0

**Published:** 2019-07-11

**Authors:** Mithra N. Hegde, Priya Gatti, Nidarsh D. Hegde

**Affiliations:** 1 Department of Conservative Dentistry & Endodontics, A. B. Shetty Memorial Institute of Dental Sciences, NITTE (deemed to be university), Mangalore, 575018 India; 2 A. B. Shetty Memorial Institute of Dental Sciences, NITTE (deemed to be university), Mangalore, 575018 India; 3 Dental speciality clinic, Mangalore, 575002 India

**Keywords:** Oral cancer, Oral conditions

## Abstract

**Introduction:**

Irradiation is known to cause oxidation process among the tissue-altering the properties of teeth leading to tissue necrosis and caries formation. Hence protection of the oral cavity is a major concern to deal with therapy side effects.

**Aim:**

Evaluation of wear resistance property of Enamel against electron beam radiation and analysing the radio protective effects of natural organic compounds.

**Materials and methods:**

Total of 36 healthy extracted human molar teeth were collected, four samples were used as control, and remaining 32 were divided into four groups (*N* = 8 each): radiation control group and three groups treated with organic compounds during radiation treatment. The enamel samples were tested for FTIR spectroscopy, XRD analysis, SEM and EDAX analysis before and after 70 Gy radiation treatment.

**Results and discussion:**

The particle size of radiation control samples had increased showing decrease in its crystallinity index. Calcium to Phosphorous ratio had also decreased along with structural changes as observed by SEM analysis. But groups treated with organic compounds has maintained tooth integrity in comparable to control groups after radiation treatment.

**Conclusion:**

Virgin coconut oil, vitamin E oil and curcumin has potential radioprotective action against radiation in protecting tissue properties. Hence, with further advanced research, these natural substances should emerge as a topical applicator during radiotherapy to oral cancer patients.

## Introduction

Oral cancer is one of the commonest cancer’s rankings sixth around the world.^1^ Ninety percent of the cases are epidermoid squamous cell carcinomas (ESCC) in the lining of the oral cavity.^2^ Alcohol, smoking and tobacco consumption are considered as risk factors elevating the levels of malignancy.^3^ While, human papilloma virus and chronic infection in the oral cavity might be the other causes for cancers in non- smokers and drinkers.^4^ The treatment of cancer involves a series of process including drugs, chemotherapy, surgery followed by radiotherapy or a combination of these therapies. However, the aggressive mode of treatment against the oncological condition has unavoidable effects on normal cells within the vicinity of exposure.^5^ It mainly affects salivary glands leading to xerostomia, muscular atropy, dental caries, fungal and bacterial infections and many more.^6^ Radiation caries is the late indirect effect observed in the patients causing irreversible damage on the dental tissues.^7^ Evidence suggest that demand for dental treatment is around 58–98% among oral cancer patient^8^ Also, dental treatments are highly recommended in prior to prevent post radiation effects, especially to extract teeth with poor prognosis having high risk of infection.^8^ Because, there are high possibility to develop caries or osteonecrosis in irradiated teeth due to softening of the tissue, plaque formation^9^ and changes in dental tissue properties decreasing surface microhardness (SMH),^7,10^ ultimate tensile strength^11^ and fracture resistance.^12,13^

Enamel, the hardest mineralized tissue, tends to degrade its anti-wearable property when subjected to chemical environment inside the mouth or in a secondary environment (in the presence of corrosive compounds). Studies conclude that the anti-wear properties of enamel significantly decrease when teeth get exposed to acidic environments^14^ or during tooth bleaching.^15^ This causes alteration in functional components of the tissue probing difficulty in mastication and changing the overall pulpal pathology. Similar reactions are also observed among patients after radiation treatment. Nevertheless, irradiation is known to cause oxidation process capable of breaking the crystalline nature of teeth. Even the micro structural organization and relative composition of the organic, mineral component of the tissue develops variations from within. Hence protection of the oral cavity is a major concern to deal with therapy side effects. The oncologists and dentists need to collaborate in a symbiotic relationship to suppress or eradicate the dreadful complications in the oral cavity.^16^ It is strongly advised by various researchers that patients must consult and receive treatment by a dentist to prevent side effects during and after radiotherapy.^17^ In addition, natural radioprotectors are extensively studied to condemn the negative effects on healthy tissues.^18^ However, very little is known about natural tooth radioprotectors and its actions. In the ancient traditional system, oil pulling technique was followed to maintain general oral health and many other plant-based compounds against allergies and infections.^19^ Hence, depending on the natural sources to combat therapy side effects will be a major milestone in “after-cancer therapy”. The present study used Coconut oil, turmeric and vitamin E oil to determine their potential role in protecting tooth integrity against Electron Beam Radiation (EBR). The proposed hypothesis of the study is that the tested compounds will effectively prevent wear resistance behaviour and protect tooth enamel against EBR.

## Materials and methods

### Tooth collection and specimen preparation

The healthy extracted human molar teeth were collected from the Department of Oral and Maxillofacial Surgery, A B Shetty Memorial Institute of Dental Sciences, Mangaluru. The consent waiver form was obtained from the institute to collect these extracted teeth samples. The experimental procedures were approved by the Human Ethics Committee of Nitte University, and the methods were carried out in accordance with the Declaration of Helsinki (2008). A total of 36 human teeth were selected for this study. They were maintained in artificial saliva (0.375 g/l calcium chloride, 0.125 g/l Magnesium chloride, 1.2 g/l Potassium chloride, 0.85 g/l Sodium Chloride, 2.5 g/l Sodium Hydrogen Phosphate, 1 g/l sorbine acid, 5 g/l carboxymethylcellulose sodium and 43 g/l sorbitol solution (70%, noncrystalline)) until the initiation of the experiment. Four samples were used as control (without radiation treatment), remaining 32 samples were divided into four groups (*N* = 8 each) based on the topical application used for protection during irradiation treatment: (1) radiation control group in distilled water, (2) test group in coconut oil, (3) test group in vitamin E oil and (4) test group in turmeric.

Out of eight samples from each group, two are used for Scanning Electron Microscopy and EDAX analysis and rest of the six samples are used for FTIR and XRD analysis.

The enamel portions of the teeth specimens (control and test sample groups) were drilled using airotar under running water to avoid the dehydration caused by local overheating. The powdered enamel samples were dried and used for the Fourier Transmission Infra-Red (FTIR) spectroscopy and X-Ray Diffraction (XRD) analysis.

### Irradiation procedure

The powdered teeth specimens (*n* = 6/group) were immersed in the solutions for Group 1,2 and 3 and layered with a turmeric in vaseline combination (polymer form) for group 4 in polypropylene Eppendorf tubes (Tarsons). FTIR and XRD testing was done at 0 Gy initially before starting the radiation treatment. Following the testing, the samples were again immersed in the solutions till they received 70 Gy of electron beam radiation using Linear Accelerator (Dual-energy photons, five energies of electrons and CT simulator) with daily exposure of 7 Gy fraction/day for ten days at Nitte Leela Narayan Shetty Memorial Cancer Institute. When they reached 35, 42, 49, 56, 63 and 70 Gy radiation, the samples were washed and dried to conduct FTIR and XRD testing at various radiation intervals. The total dosage of radiation and the course of therapy were followed as per the module for oral cancer patients to simulate the clinical situation.^20^ All the sample tubes were immersed in water to distribute the radiation dosage equally throughout the medium. After irradiation, the specimens were thoroughly rinsed with deionized water and then tested.

### Test period

The samples were tested for their change in the properties at various radiation intervals. Four tooth samples were used as control and tested without radiation treatment. The remaining six samples were tested for FTIR and XRD after receiving 0, 35, 42, 49,56,63 and 70 Gy Electron beam radiation dose. The samples were further processed and evaluated

### FTIR analysis

FTIR spectrometric analysis of the enamel was performed using a spectrometer (IRPRestige-21, Shimadzu, Japan) under the supervision of expert supervisor at Mangalore University. The spectra were recorded in the range of 400–4000 cm^−1^ at a 4 cm^−1^ resolution. The samples were positioned against the diamond crystal of the FTIR unit and pressed with a force gauge at a constant pressure to facilitate contact. The spectra of the enamel before and after irradiation at intervals were obtained. Data were recorded and analyzed with Origin Pro 8.0 SRO software (Northampton, USA). The band between 500 and 885 cm^−1^ represents CO3^2−^v2, while the band between 885 and 1090 cm^−1^ provided information about PO4^3−^ v1, v3. After normalization, the ratio of the integrated areas of the CO3^2−^ v2 peak to the PO4 ^3−^v1, v3 peak (C: M value) were measured.^21^

### X-Ray diffraction

The crystalline nature of the enamel specimens was evaluated before and after irradiation treatment at various study interval using X’pert XRD (X’pert PRO, Panalytical, Netherlands) with CuKα radiation source at 35 kV/25 mA. Data were obtained in the 2θ range of 10°–70°. Both, crystallinity and grain size were calculated. The crystal sizes were calculated using Scherrer’s formula^22,23^ as follows: D = 0.89 λ /βcosθ, where λ represents the wavelength (CuKα), θ is the peak diffraction angle that satisfies Bragg’s law for the hkl plane and β is the width of the diffraction profile. The CIXRD index of XRD patterns was measured using several peaks located at the proximity to the highest peak in the graph as suggested by Pearson et al.^19^ The reflections chosen with reference standard hydroxyapatite were (211), (202) and (300) located between 30 and 35° of the 2- theta angle. The height of the highest peak corresponding to (211) was measured from the baseline fit and heights of the other peaks were from the top to valley separating it from the corresponding peak.

The CIXRD value is given by:

$$\left( {{\mathrm{CI}}} \right){\mathrm{XRD}} = \frac{{{\mathrm{a}}\left( {{\mathrm{300}}} \right) + {\mathrm{b}}\left( {{\mathrm{202}}} \right)}}{{{\mathrm{h(211)}}}}$$

### SEM analysis

The eight samples were marked at a point on its enamel surface using a diamond marker pen. Morphological changes observed at these marked regions at various intervals using SEM (Carl Zeiss- Fesem, Oxford Instruments, EDS) to reveal the deformation and fracture patterns. SEM edax were performed to analyze the compositional change at the region on the enamel at different irradiation intervals. The results of compositional change for Calcium, Phosphate, Oxygen and sodium expressed in terms of percentage of atomic mass and weight.

### Statistical analysis

Data were analysed using Statistical Package for the Social Sciences (SPSS) version 16.0 software (SPSS Inc, Chicago, IL, USA). A paired *t*-test was used to compare crystallinity, crystal size, C: M ratio and Compositional change before and after the irradiation treatments at various intervals. A p- value less than 0.05 (*p* < 0.05) was considered statistically significant.

## Results

### XRD results

**Fig. 1:**
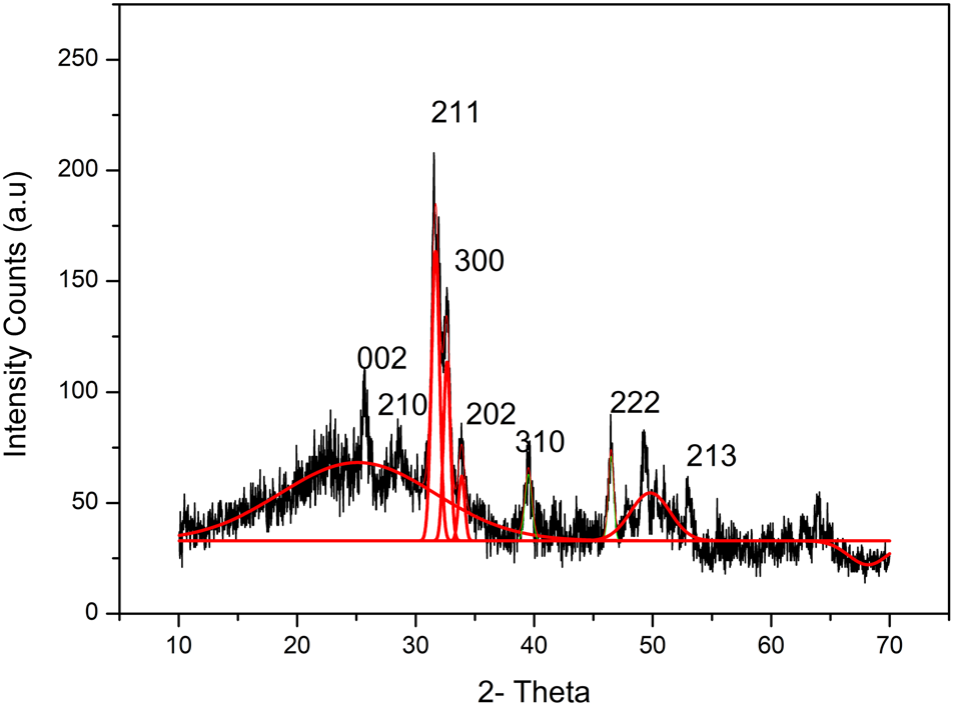
X-ray diffraction patterns of sound enamel powder (control group—non radiated)

**Fig. 2:**
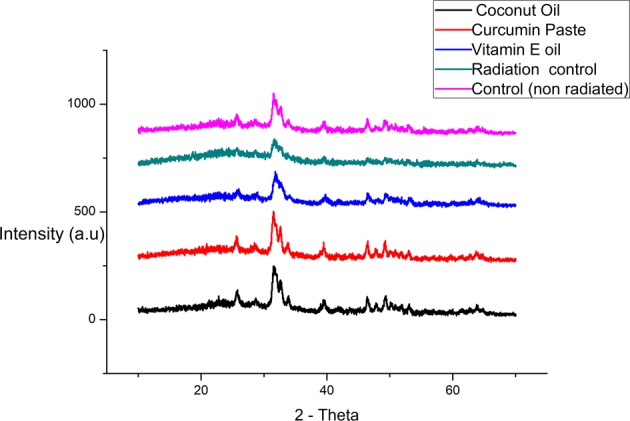
X-ray diffraction patterns of sound enamel powder after treatment with 70 Gy electron beam radiation maintained at different natural protective agents **a** virgin coconut oil, **b** curcumin paste, and **c** vitamin E oil. **d** Radiation control group in distilled water, **e** non radiated group for comparison

X-ray diffraction (XRD) spectra of the enamel before and after irradiation are shown in Figs. 1 and 2. The hkl Miller indices assigned correspond to the standard hydroxyapatite diffraction pattern (JCPDS 86–0740). The test enamel XRD patterns were compared with that of reference hydroxyapatite, and the peaks were found to be consistent. Meanwhile, the powder XRD analysis indicated that irradiation had decreased the crystallinity of enamel and enlarged the crystal size (Table 1). However, the protective mechanism was exhibited by the natural agents retaining the crystallinity of the enamel. Virgin coconut oil appeared as the best natural product among the studied materials followed by curcumin and vitamin E oil. Table 2 shows the particle size and crystallinity Index as measured from the Figs. 1 and 2 using squerrer equation and millers’ indices. It has been noted that the particle size of the radiated sample maintained in distilled water had increased showing a decrease in its crystallinity index. The CI XRD values of samples maintained in protective medium showed better crystalline Index than compared to radiation control group.

**Table 1:** Particle size calculated after Irradiation (70 Gy) using the Scherrer equation and the (CI) XRD based on the miller Indices and peak height from the respective graphs

Sample	Particle size (nm)	Crystallinity index CI(XRD) ± 0.01
Control (non radiated)	22.9	1.14
Radiation control	25.4	1.03
Virgin coconut oil	22.6	1.18
Vitamin E oil	23.1	1.16
Curcumin	22.7	1.17

**Table 2:** Organic compounds used in the study

SI. No	Organic compound list	Company name	Composition
1	Virgin coconut oil	Ayush Ltd, India	Coconut oil- cold pressed
2	Vitamin E capsules USP, Evion 400	Merck Consumer Healthcare Ltd	Vitamin E
3	Curcumin (Code 97461)	Sisco Research Laboratories Pvt. Ltd.(SRL) - India	Curcumin

### FTIR analysis

**Fig. 3:**
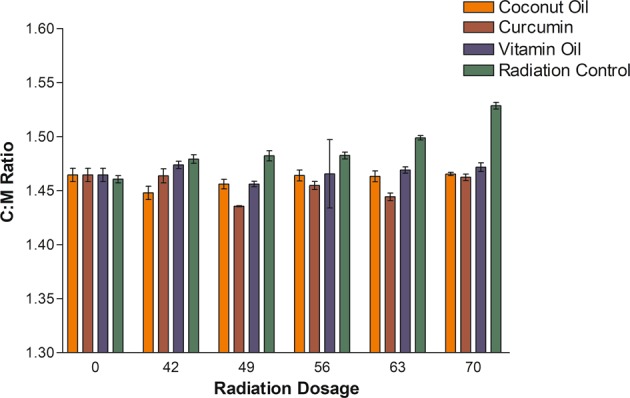
Graphical representation of C:M Ratio of Tooth enamel at various radiation intervals under specific protective medium

**Fig. 4:**
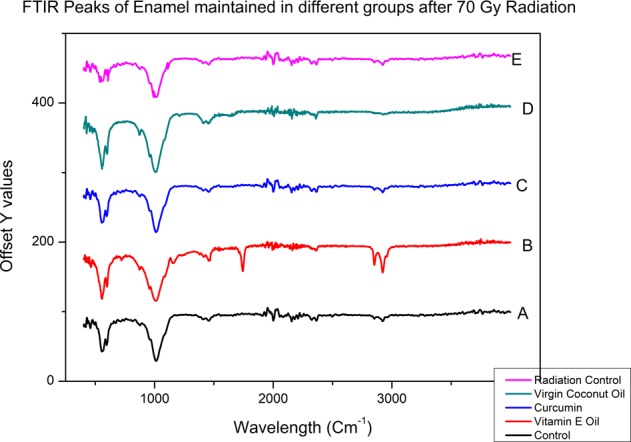
FTIR spectra of enamel powder at different subjective mediums after radiation 70 Gy treatment **a** control (non radiated), **b** vitamin E oil, **c** curcumin paste, **d** virgin coconut oil, and **e** radiation control

The representative Fourier transform infrared spectroscopy (FTIR) spectra were measured for its absorbance in all the enamel test groups. The integrated area of CO^2−^ v2 and PO^3−^v1, v3 peaks were measured. The radiation control group showed a significant decrease in peak size and area in the enamel components after 70GY irradiation treatment when compared to the non-radiated group. The decrease in PO_4_^3−^ v1, v3 integrated area was higher than the CO ^2−^ v2 area. The results of the paired *t*- test revealed a significant increase in the carbonate: mineral ratio (C: M) after irradiation in the control groups and a significant decrease in the ratio among the protective media groups (Table 3). The test groups did not show a major difference at all the radiation interval periods maintaining insignificant stable mineral ratio thereby improving area retention at all the experimental periods. (Figs. 3 and 4)

**Table 3:** One Way ANOVA of C:M ratio of Tooth enamel before and at various radiation intervals in relation to subjective medium

Radiation dosage (Gy)	Coconut Oil (*n* = 6) ± 0.05	Curcumin (*n* = 6) + 0.05	Vitamin oil (n = 6) + 0.05	Radiation control (n = 6) + 0.05	*P* Value
0	1.46	1.46	1.46	1.46	0.769
42	1.44	1.46	1.48	1.48	<0.001
49	1.45	1.44	1.45	1.48	<0.001
56	1.46	1.45	1.45	1.48	0.282
63	1.46	1.44	1.47	1.5	<0.001
70	1.46	1.46	1.47	1.52	<0.001

### SEM and EDAX analysis

**Fig. 5:**
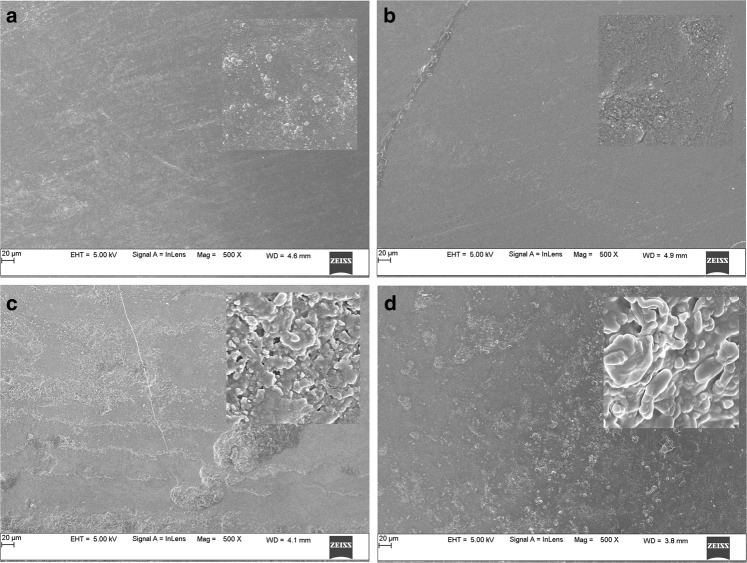
Scanning Electron Microscopy images of Human tooth enamel at 35 Gy radiation period, Showing pattern at 500x and 10000x (right corner image) magnification **a** control group, **b** Enamel in virgin coconut oil, **c** vitamin E oil, and **d** curcumin paste

**Fig. 6:**
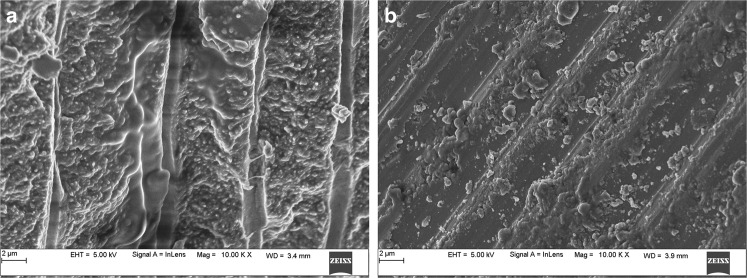
Scanning Electron Microscopy images of Human tooth enamel of control Group showing clear prisms surrounded by interprismatic regions before Radiation at 0 Gy, **a** and disrupted prismatic structures after subjecting to 70 Gy Radiation treatment, **b** observed under 10000x magnification

**Fig. 7:**
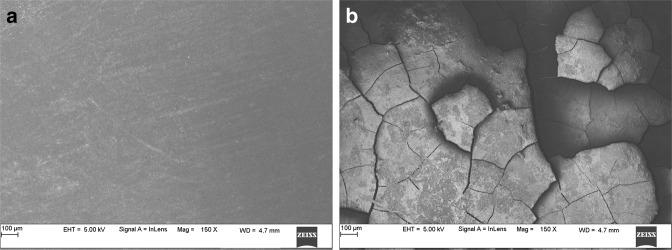
Scanning Electron Microscopy images of Human tooth enamel of control Group showing no evident cracks before Radiation at 0 Gy, **a** and multiple cracks after subjecting to 70 Gy Radiation treatment, **b** observed under 150x magnification

**Fig. 8:**
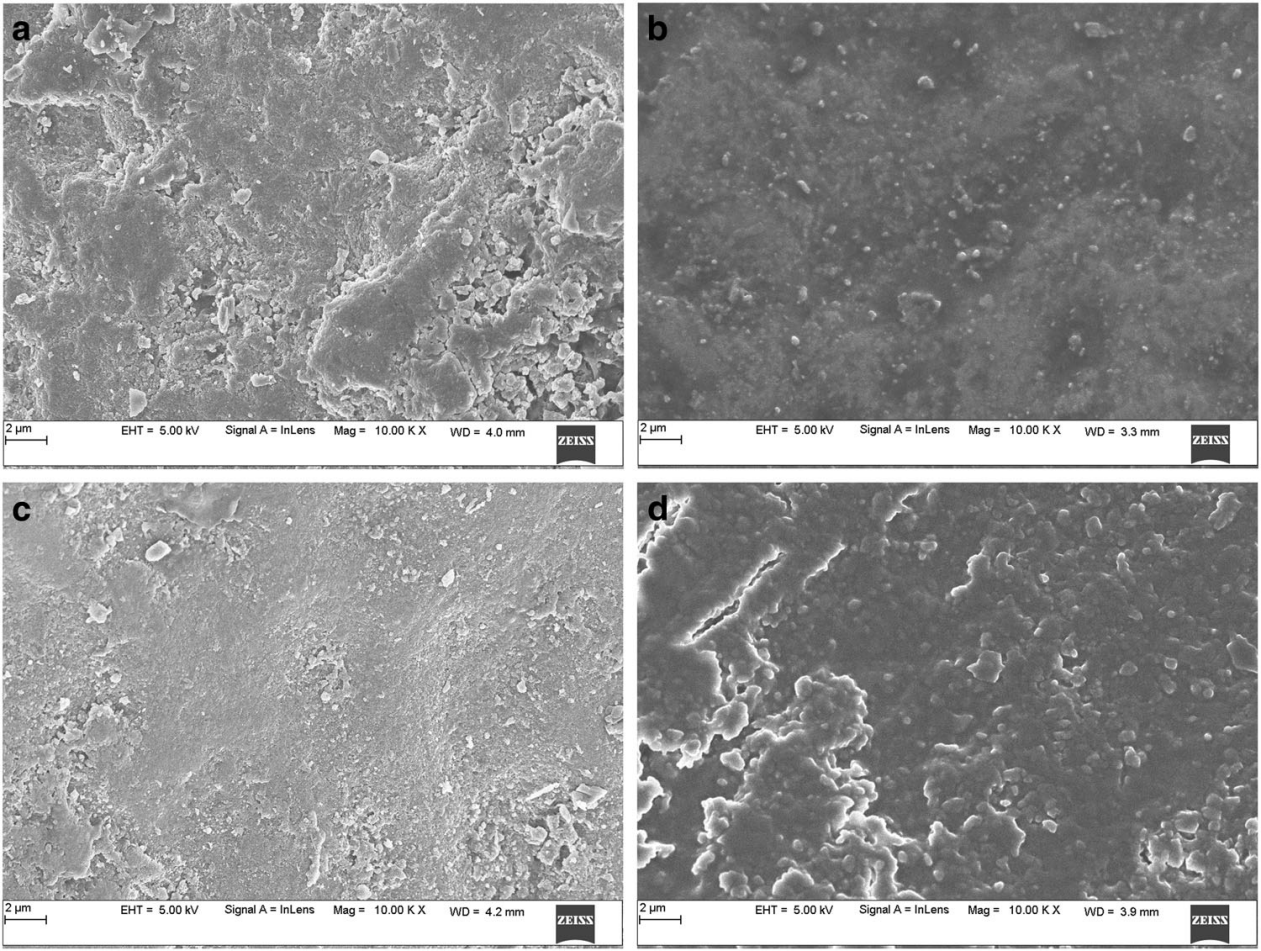
Scanning Electron Microscopy images of Human tooth enamel at 70 Gy radiation period, Showing disturbance and amorphous powders in the pattern at 10000x magnification in **a** Control Group and limited variation in the test groups, **b**) enamel in virgin coconut oil, **c** vitamin E oil, and **d** curcumin paste

The Scanning Electron Microscopy (SEM) images of normal perpendicular sectioned enamel showed clear prisms surrounded by inter prismatic region before radiation. Distinct differences existed in the morphology of enamel in the same portion after radiation with disrupted prismatic structure, which appeared as amorphous substances in all radiated enamel specimen. No debris was found before irradiation; amorphous substance appeared after irradiation treatment (Figs. 5–8).The structural deformities were seen in the test groups after radiation treatment with slightest modifications at various radiation intervals. The EDAX analysis detected calcium (Ca), phosphorus (P) and oxygen (O) elements in the tooth sample. Oxygen had the highest percentile compared to other elements, with 331.12% and 52.68%, respectively before radiation treatment. After radiation treatment, the Oxygen content decreased drastically to 236.82% and 48.71%, respectively. The phosphate to calcium ratio also varied at the end of the treatment procedure, but the test groups showed an insignificant difference in oxygen, phosphate and calcium content in comparison to the control Group (Table 4). One-way ANOVA was performed to test between and within group differences for oxygen, phosphorous and calcium levels. There was a significant difference observed with a *p*-value < 0.001. Further, multiple comparisons between the groups were conducted using Tukey’s post hoc and Dunnets *t*-test. Tukey’s post hoc test revealed a significant difference between the test values of Radiation control and test groups. Based on the Dunnets *t*-test, we determined that there is no significant difference between the control and test groups, but there is a significant mean difference observed between radiation control and control groups for oxygen and phosphorous atomic mean%. (Table 5). For calcium levels, the mean difference was not statistically significant with a *p*-value = 0.340.

**Table 4:** Atomic % of Oxygen, phosphorous and Calcium content in tooth samples before and after radiation as per SEM EDAX analysis

Groups	Oxygen (atomic %)		Phosphorous (atomic %)		Calcium (Atomic %)	
	Mean	Std. deviation	Mean	Std. deviation	Mean	Std. deviation
Control	53.3800	.68088	8.7100	.40841	10.9500	.24269
Radiation control	48.9433	.85424	7.2500	.24000	9.8533	.33081
Virgin coconut oil	52.1700	.66731	10.6467	.25166	13.8967	.77694
Vitamin E oil	52.4567	.34298	10.4833	.68296	13.3167	2.13001
Curcumin	52.4933	1.21162	9.1933	.78806	11.5633	1.36676
Total	51.8887	1.72063	9.2567	1.36407	11.9160	1.85058

**Table 5:** Multiple comparisons within group analysis by Dunnet’ *t*-Test

	I	J	Mean difference (I–J)	Std error	Sig.a	95% confidence interval
Oxygen (atomic %) (<control)^a^	Radiation control	Control	−4.4*	0.65	<0.001	−2.820
	Virgin coconut oil	Control	−1.2	0.65	0.131	0.407
	Vitamin E oil	Control	−0.92	0.65	0.240	0.693
	Curcumin	Control	−0.89	0.65	0.257	0.730
Phosphorous (atomic %) (<control)^a^	Radiation control	Control	−1.46*	0.43	0.011	−0.404
	Virgin Coconut oil	Control	1.93	0.43	1.00	2.992
	Vitamin E oil	Control	1.77	0.43	1.00	2.83
	Curcumin	Control	0.48	0.43	0.980	1.54
Calcium (atomic %) (<control)^a^	Radiation control	Control	−1.09	0.98	.340	1.32
	Virgin coconut oil	Control	2.94	0.98	1.000	5.36
	Vitamin E oil	Control	2.4	0.98	0.999	4.78
	Curcumin	Control	0.61	0.98	0.939	3.03

*The mean difference is significant at the 0.05 level

^a^Dunnett *t*-tests treat one group as a control, and compare all other groups against it

## Discussion

Radiotherapy is a basic treatment protocol that is widely used to manage head and neck oral cancer. The tribological properties^24^ of enamel play an important role in the normal functioning of the tooth. Therefore, understanding of the wear behaviour of irradiated tooth enamel is of great interest in determining the quality and integrity of the tooth sample. In the present study, we reported that Electron beam radiation eventually reduces the wear resistance property of enamel on subjecting to 70 Gy Radiation dose treatment. Nevertheless, the organic compounds were found capable in protecting the tissue properties at all the study intervals. Hence the proposed hypothesis has been proved in this study. Among different techniques, XRD was used to analyze the changes in the crystalline structure of human tooth enamel^25^ during and after complete radiation treatment. It was found that irradiation induced a reduction in enamel crystallinity and enlarged crystal size (Table 3). The crystallinity of enamel has been widely reported to play an important role in its mechanical properties.^26,27^ Given that, the reduction in crystallinity indicates decreased mechanical properties, such as a reduction in hardness, which has been proven previously in our studies.^28^ These changes usually occur due to demineralization process, which starts after the pH of the oral cavity drops below the “critical value” (5.5 for hydroxyapatite), causing disintegration of the calcium phosphate ions in the hydroxyapatite crystals.^29^ Sound enamel with a high crystallinity shows excellent mechanical and anti-wear properties. On the other hand, the groups treated with virgin coconut oil, vitamin E oil and curcumin showed better crystallinity compared to that of the radiation control group. Oral hygiene plays a major role in preserving the properties of oral tissues. Oil pulling was one such method used in traditional medicine to maintain good oral and general health.^30^ It is true that during the process oil forms a protective layer and maintains the favourable pH to prevent demineralization.^31^ Further, Curcumin (diferuloylmethane), an odourless yellow pigment isolated from the rhizome of turmeric (Curcuma longa) is known for skin protection and marked application in cosmetic industry. The curcumin usage in the study also showed positive results, indicating its protective action against Electron beam radiation.^19^ Also, it has been found that topical application of curcumin in epidermis of CD-1 mice significantly inhibited UVA-induced ornithine decarboxylase ornithine decarboxylase (ODC) activity and prevented apoptotic changes in human epidermoid carcinoma A431 cells.^32^ To complement the information obtained by X-ray diffraction analysis, enamel sample was further characterized by Fourier Transmission infrared (FTIR) spectroscopy. This technique examines the inorganic materials and quantitatively measured the alterations in the composition of mineralized tissue.^33^ The results showed that the integrated area of PO43− v1, v3 significantly decreased and the C: M ratio dramatically increased in the radiation control group after radiation treatment. This is the indication that irradiation resulted in a reduction of the mineral content of the enamel.

On the other hand, the organic compound treated samples depicted significantly lower C: M ratio compared to that of the radiation control group. Also, there exists a positive correlation between microhardness and the mineral content of the tooth enamel.^34,35^ Hence the reduction in mineral content of enamel after irradiation is the cause for the decrease in micro hardness property of the enamel.^36^ But the test group compounds were successful in maintaining the nearly stable mineral content ratio during and after radiation therapy.

**Fig. 9:**
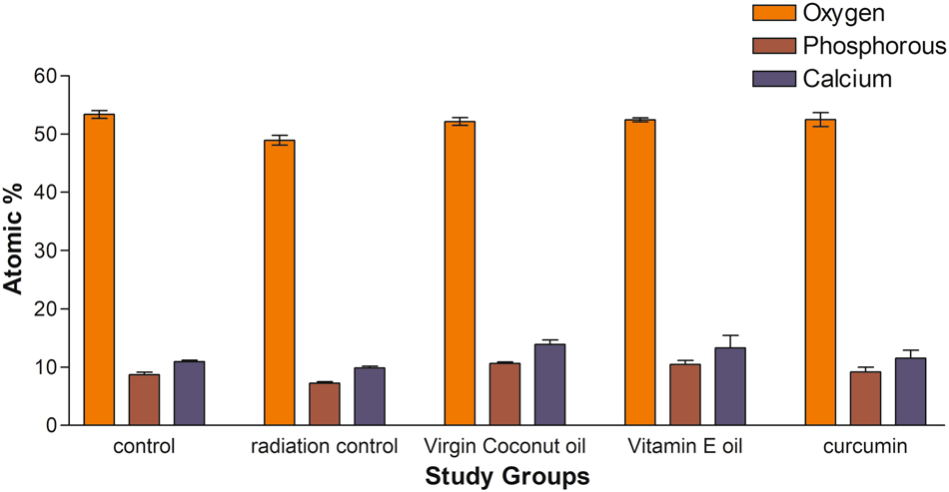
Graphical representation of SEM- EDAX data for Atomic % of oxygen, phosphorous, and calcium content in tooth enamel samples before and after radiation

The SEM images of the enamel portion at various radiation intervals are shown in the Figs. 1–4. The enamel of the non-irradiated teeth displayed well-organized prisms, surrounded by interprismatic regions.^22^ Whereas the radiation control group after 70 GY radiation shows structure disorganization with considerable longitudinal crack and few other smaller cracks. With increasing doses of irradiation, a progressive change was also observed in the prismatic structure of the enamel, impairing the identification of the prisms.^23,37^ The electron micrographs of the enamel exposed to therapy on covering the sample with radioprotective groups revealed a progressive change in the enamel surface at different radiation intervals. In addition, changes in the structure of radiated sample group had no major difference from the non-irradiated enamel; and interprismatic regions were more clearly observed compared to that of the Radiation control groups. Moreover, data obtained from SEM EDAX system detected calcium (Ca), phosphorus (P), and oxygen (O) elements in the tooth sample. Oxygen had the highest percentage of atomic and weighting compared to other elements before radiation treatment. The level of oxygen composition showed a linear decrease as the radiation dose was increased to 70 Gy (Fig. 9). This is due to the thermal energy released by the radiation source, which can reduce water content in the tissue initiating the oxidation process. Calcium and Phosphorous are the next main elements found in the teeth. The Calcium-Phosphorous ratio had also tamed after the radiation treatment, but the Experimental groups prevented the oxidation process maintaining the oxygen content to nearly normal to that of the control group. Overall, the present in vitro study indicates that the changes in crystallography and composition of enamel after irradiation treatment may contribute to reduction in the enamel’s wear resistance properties. To avoid this alteration in mechanical and morphological properties, an effective protective treatment plan should be developed before, during, and after irradiation treatment. Use of remineralizing agents or gels has been suggested to prevent the post-treatment side effects.^38^ But the use of natural agents to protect the teeth during treatment has not been implemented. Therefore, this study serves an evidence to understand the effects of radiation on dental tissues and explains the need to develop measures in preventing oral complications. The study observes virgin coconut oil, vitamin E oil and curcumin to have potential radioprotective features against electron beam radiation in conserving the properties of teeth. The study recommends the use of these natural substances as topical applicator during treatment to reduce the side effects of radiotherapy. Further research requires to be conducted to formulate these compounds and standardise the protocols for clinical usage.^38^

## Conclusion

The study findings have provided evidence to justify that radiation treatment up to 70 Gy is causing damage to the enamel tissue property where it loses its mechanical strength, undergoes compositional change along with structural variations as studied by XRD, FTIR and SEM EDAX analysis. The topical applications with organic compounds from natural source during radiation treatment have preserved the tissue integrity of teeth. This widens the scope for future research to develop a natural protective compound to be applied during radiotherapy.
